# Atrophic Vaginitis in Breast Cancer Survivors: A Difficult Survivorship Issue

**DOI:** 10.3390/jpm5020050

**Published:** 2015-03-25

**Authors:** Joanne Lester, Gaurav Pahouja, Barbara Andersen, Maryam Lustberg

**Affiliations:** 1 Clinical Research Nurse Practitioner, Division of Surgical Oncology, The Ohio State University, Columbus, OH 43210, USA; 2 Department of Psychology, The Ohio State University, Columbus, OH 43210, USA; E-Mail: andersen.1@osu.edu; 3 Comprehensive Cancer Center, Arthur G. James Comprehensive Cancer Hospital and Richard J. Solove Research Institute, Columbus, OH 43210, USA; E-Mail: Maryam.lustberg@osumc.edu; 4 Northeast Ohio Medical University, Rootstown, OH 44272, USA; E-Mail: gpahouja@kent.edu; 5 Division of Medical Oncology, The Ohio State University, Columbus, OH 43210, USA; 6 Stefanie Spielman Comprehensive Breast Center, 1145 Olentangy River Rd, Columbus, OH 43212, USA

**Keywords:** atrophic vaginitis, breast cancer, vaginal dryness, dyspareunia, vaginal estrogen, survivorship, chemotherapy-induced ovarian failure, endocrine therapy, breast cancer, non-hormonal vaginal therapy

## Abstract

Management of breast cancer includes systematic therapies including chemotherapy and endocrine therapy can lead to a variety of symptoms that can impair the quality of life of many breast cancer survivors. Atrophic vaginitis, caused by decreased levels of circulating estrogen to urinary and vaginal receptors, is commonly experienced by this group. Chemotherapy induced ovarian failure and endocrine therapies including aromatase inhibitors and selective estrogen receptor modulators can trigger the onset of atrophic vaginitis or exacerbate existing symptoms. Symptoms of atrophic vaginitis include vaginal dryness, dyspareunia, and irritation of genital skin, pruritus, burning, vaginal discharge, and soreness. The diagnosis of atrophic vaginitis is confirmed through patient-reported symptoms and gynecological examination of external structures, introitus, and vaginal mucosa. Lifestyle modifications can be helpful but are usually insufficient to significantly improve symptoms. Non-hormonal vaginal therapies may provide additional relief by increasing vaginal moisture and fluid. Systemic estrogen therapy is contraindicated in breast cancer survivors. Continued investigations of various treatments for atrophic vaginitis are necessary. Local estrogen-based therapies, DHEA, testosterone, and pH-balanced gels continue to be evaluated in ongoing studies. Definitive results are needed pertaining to the safety of topical estrogens in breast cancer survivors.

## 1. Introduction

Improved treatment and screening for female breast cancer has resulted in higher survival rates over the past two decades with five-year survival rates today as high as 90% [1]. As a result, there are nearly 3 million breast cancer survivors living in the United States today [1]. Recommended systemic therapies for the management of breast cancer may include chemotherapy or endocrine therapy, which can cause a variety of short- or long-term symptoms that can impair quality of life [2,3,4]. One such treatment side effect is atrophic vaginitis, caused by decreased levels of circulating estrogen to urinary and vaginal receptors [5,6,7]. Atrophic vaginitis is a survivorship issue that affects nearly 70% of postmenopausal breast cancer survivors as compared to 50% of postmenopausal women without breast cancer [8,9,10].

## 2. Symptoms of Atrophic Vaginitis

Atrophic vaginitis is prevalent in menopausal women, but more so in postmenopausal breast cancer survivors [8,9,10]. Symptoms of atrophic vaginitis include vaginal dryness, dyspareunia, irritation of genital skin, pruritus, burning, vaginal discharge, and soreness [11,12,13,14,15,16,17,18,19,20]. Atrophic vaginitis can disrupt sexual activity, and lead to problems such as pain with vaginal penetration (dyspareunia), decreased lubrication, and fear of pain with sexual activity [12]. Symptoms of atrophic vaginitis can be disruptive in women with, or without partners, with or without sexual activity [12]. Premature menopause with associated symptoms in young breast cancer patients (e.g., less than or equal to 40 years of age) may have a profound negative impact on quality of life secondary to sexuality and intimacy changes [4]. Increasingly, women of all ages are seeking to preserve their sexual function and improve their sexual quality of life [12,21,22]. Thus, atrophic vaginitis as a survivorship issue impacts women of all ages.

Discernible symptoms of atrophic vaginitis typically occur within 4–5 years after a woman’s last menstrual cycle, and can increase in prevalence and severity over time [23]. In women who undergo menopause at an accelerated rate (e.g., post-surgical bilateral oophorectomy or drug-induced such as with chemotherapy), symptoms of atrophic vaginitis can occur sooner and be more severe than is found among those with a normal, paced menopause [23] yet is often overlooked and underdiagnosed [24].

Atrophic vaginitis is prevalent as suggested in a study of women with and without breast cancer. [10] Complaints of vaginal dryness were 67% and 49% and fear of pain with vaginal penetration, 31% and 19%, respectively [10]. Women with breast cancer complained of irritation from toilet tissue (21%) as compared to those women without breast cancer (9%); both groups complained of external genital itching (30% and 29% respectively), vaginal itching (28%) and vaginal odor (29%) [10]. While not all women develop atrophic vaginitis [10], those that do have significant symptoms. Despite these bothersome symptoms, few women discuss them with physicians or seek gynecological care [12,25], in part due to embarrassment, lack of knowledge, and an awareness of menopausal changes. Therefore, it is imperative that clinicians ask both partnered and unpartnered patients about potential physical changes and alterations that can be associated with atrophic vaginitis [12].

## 3. Diagnosis of Atrophic Vaginitis

The diagnosis of atrophic vaginitis is confirmed through patient-reported symptoms and gynecological examination of external structures, introitus, and vaginal mucosa. The clinician must be cognizant that the visible signs and reported symptoms may not be analogous [2] Subjective symptoms can initially be evaluated through a self-report questionnaire [12] and further discussed with clinicians.

Several objective findings on physical exam may be relevant. The gynecologic examination may reveal sparse vulvar hair, decreased subcutaneous fat within the mons pubis and labia majora, reduced volume of labia minora, retracted clitoris, and inadequate vaginal lubrication with a dry, shiny and pale introitus [5,7,8,14]. In research studies, samples of vaginal secretions can be obtained to determine vaginal pH, presence or absence of yeast, and parabasal cell level. The parabasal cell level is used to define the vaginal maturation index (VMI), which is the proportional relationship between parabasal, intermediate, and superficial cells in the vaginal squamous epithelium [26]. In an estrogen rich environment, superficial cells are dominant and thicken the epithelium. However, when estrogen levels are reduced, parabasal cells are highly prevalent with fewer intermediate and superficial cells [7,26]. VMI can be used as an indicator of the degree of vaginal atrophy. A VMI of ≤ 49 is suggestive of little or no estrogenic effect, 50–64 indicates a moderate estrogenic effect, and 65–100 supports a dominant (e.g., normal premenopausal) estrogenic environment [7].

## 4. Biological Changes Due to Atrophic Vaginitis

Systemic loss of estrogen results in physiological and structural modifications within the genital structures and vaginal mucosa. Post-menopausal estrogen depletion precludes reduced transudation through the vaginal epithelium and reduced cervical gland secretions [7,24,27]. Deterioration of tissue, decrease in blood flow, loss of elasticity, decreased rugae, thinning of tissues and epithelium, and increased pH are all consequences of low estrogen receptor binding [14,28]. Collagen fibers combine while elastin fibers divide in the dermal layer causing a loss of mucosal elasticity [12] with a shortening and narrowing vagina. The thinning of tissues and epithelium in the vagina are a result of fewer squamous cells in secretions. The vaginal mucosa has reduced glycogen content and lack lactobacilli which convert glycogen into lactic acid to maintain a healthy vaginal pH in the range of 3.5–4.5 [7]. As glycogen levels fall, the amount of lactobacilli decreases and vaginal pH increases to the range of 5.0–7.5 [7,10,11,16]. Such atrophic changes therefore predispose women to symptoms and vaginal infections as the base environment enables infection from pathogenic bacteria such as staphylococci and group B streptococci [7].

The decreased blood flow is in part responsible for increased vaginal dryness as vaginal secretions are a byproduct of surrounding blood vessels. As the volume decreases from various structures in the external genitalia, further reduction in secretions occur. The cells that are sloughed are not robust and therefore contain minimal amounts of secretions [7]. In summary, atrophic vaginitis is a result of multiple changes in the external genitalia and internal mucosa with inflammation, overgrowth of pathogens, and a resultant acidic environment [2,5,7,11,12].

## 5. Effects of Breast Cancer Treatment

### 5.1. Chemotherapy

The majority of women with breast cancer receive systemic treatment in the form of chemo-, hormonal-, or biologic therapies to reduce their risk of systemic disease. Although these therapies have significantly improved clinical outcomes, they can lead to biological changes that affect long-term vaginal health and impact quality of life in survivors. Pre- and post-menopausal women can experience symptoms of estrogen deprivation including atrophic vaginitis [3,4,5,6,7,8] at higher rates than age-matched women without breast cancer [10]. In a cohort of premenopausal breast cancer survivors receiving chemotherapy, vaginal dryness was reported by 23.4% of women [29]. Postmenopausal women can also experience increased or recurrent symptoms of estrogen deprivation, depending on the amount of endogenous estrogen circulating in their system, including estrogen produced by the adrenal glands and estrogen stores in body fat.

Chemotherapy can promote ovarian failure (CIOF). The use of chemotherapy during the first year after diagnosis of breast cancer significantly increases the risk of CIOF [7,30,31,32]. CIOF occurs secondary to chemotherapeutic agents which cause follicular destruction [4,5,6,12,30,32,33]. Consequently, decreases in the levels of estrogen and progesterone are observed. Forty- and fifty-year-old women undergoing chemotherapy were found to have an increased risk of developing CIOF (e.g., 40% and 90%, respectively) [32]. In contrast, the risk of premature ovarian failure in healthy age-matched forty- and fifty-year-old women was less than 5% and 20%, respectively [32].

### 5.2. Endocrine Therapy

Women with breast cancer frequently receive systemic endocrine therapy as approximately 70%–80% of all breast cancers are considered to be estrogen receptor positive [34]. Endocrine therapy is extremely successful in suppressing circulating estrogen, an effect desired for efficacy [34,35]. Endocrine therapies including aromatase inhibitors and selective estrogen receptor modulators (SERMs); both classes of drugs can trigger the onset of atrophic vaginitis or exacerbate existing symptoms [13,16,31,36].

- Aromatase inhibitors: Aromatase inhibitors (AIs) are frequently prescribed for postmenopausal breast cancer patients [37,38,39,40,41,42,43]. Commonly reported side effects include vaginal dryness and decreased libido [44]. These drugs inhibit the activity of the enzyme aromatase that is utilized to convert androgens to estrogens [35]. Multiple clinical trials have shown that AIs have better clinical outcomes as compared to SERMs; thus they have become the standard of care [16,31,37,38,39,40,41,42,43]. The third generation of AIs available for use can terminate aromatase activity by more than 95% and significantly reduce plasma concentrations of estrogen from a high of 20 pmol/L to a low of 3 pmol/L, or less [44]. These changes explain the etiology of vaginal dryness in breast cancer survivors. The increasing use of AIs over SERMs suggests that more women may experience new or increased atrophic vaginitis [16,31,44] than when tamoxifen alone was used. The severity of menopausal side effects including atrophic vaginitis may compromise compliance with AIs over time [6]. AIs are only indicated for use in postmenopausal women, although often AIs are prescribed for premenopausal women in conjunction with a gonadotropin-releasing hormone agonist (GnRH-a) that will induce a menopausal state [38,39].
- Tamoxifen: A traditional class of endocrine therapies includes selective estrogen receptor modulators (SERMs). Tamoxifen has been the most widely used SERM and continues to be prescribed for premenopausal women with estrogen receptor positive tumors [38]. Tamoxifen is primarily metabolized by CYP2D6 and CYP3A4 enzymes to form endoxifen which binds estrogen receptors to lock activity [35,45]. Tamoxifen acts as an antagonist to estrogen positive breast cancer cells, although it often acts as an agonist to α estrogen receptors in the vagina. Hence tamoxifen provides a quasi-estrogenic effect on the vulva and vagina and increases vaginal secretions without the presence of estrogen [44]. Due to this estrogenic effect, the incidence rate of vaginal dryness in tamoxifen is only 8% as compared to 18% with AIs [16,31,44]. Therefore, this effect may inhibit the onset of atrophic vaginitis and actually improve existing vaginal dryness induced by chemotherapy or menopause.

## 6. Management of Atrophic Vaginitis

Atrophic vaginitis can be difficult to manage in symptomatic women, and even harder to manage in symptomatic breast cancer survivors due to restrictions in the use of hormones.

### 6.1. Lifestyle Modifications

First-line therapies include lifestyle adjustment and non-hormonal treatments which may decrease symptoms of atrophic vaginitis [46]. Prevention of atrophic vaginitis may not be a realistic goal, but prevention of the degree and extent of physical changes may be possible. Smoking cessation may decrease atrophic effects due to increased capillary refill [7,44,47]. Regular coitus and other sexual activities such as cuddling and masturbation improve blood flow and vaginal pH [7]. Vaginal penetration with lubricated fingers or vaginal dilators may prevent fibrotic changes and gently stretch tightened vaginal walls. Stress management may be helpful to decrease the stress associated with uncomfortable introitus insertion and fear of painful intercourse [48,49]. Scented hygiene products should be avoided as they may reduce normal vaginal flora.

### 6.2. Non-Hormonal Therapies

Lifestyle modifications measures alone are usually insufficient to significantly improve atrophic vaginitis in breast cancer survivors. Non-hormonal vaginal therapies may provide additional treatment options to alleviate or improve vaginal dryness, irritation and itching by increasing vaginal moisture [28,48,49]. Non-hormonal therapies are not able to reverse atrophy once it occurs. Non-hormonal therapies include vaginal moisturizers and lubricants and avoidance of perfumed soaps and toilet tissue, rubber products, synthetic garments including panties, and certain fabric softeners [15].

### 6.3. Vaginal Moisturizers

Vaginal moisturizers intend to replace normal vaginal secretions, although must be used on a regular basis to be effective [49]. In some studies, polycarbophil-based non-hormonal moisturizers have been shown to be more effective than lubricants and even as effective as vaginal estrogen creams in improving vaginal moisture, fluid volume, pH, and elasticity and reducing dryness, itching, and dyspareunia [50,51,52,53]. Polycarbophil, the active ingredient in Replens^©^, is an acidic bioadhesive polymer than can carry up to 60 times its mass in water to create a moist atmosphere by binding to squamous cells in the vagina [50,51,52]. Subsequently, the release of water and electrolytes occurs to induce vasodilation leading to improved hydration [50,51,52]. In addition, the acidic nature of the polymer helps to improve pH of the vagina. This effect is not sustained over time unless the moisturizer is used on a regular basis [51,52].

### 6.4. Vaginal Lubricants

Lubricants are shorter-acting than moisturizers and have no effect on vaginal pH or underlying moisture content due to the ingredients and manufacturing of the product. These products are designed to be applied during sexual activity, with direct application to the external genitalia, vaginal introitus, and vaginal mucosa. Lubricants reduce friction during sexual activity as well as irritation caused by clothing. While these interventions may improve the symptoms of atrophic vaginitis, in many cases non-hormonal therapy may not be sufficient [53] to completely eliminate pain or discomfort.

Lubricant formulations are water-, glycerin-, or silicone-based products that provide lubrication to improve genital or vaginal dryness. Products that contain glycerin (e.g., Astroglide© or KY Extended©) may provide improved comfort during sexual activity as compared to water-based products (e.g., KY jelly©), although they do not provide long-term moisture in the manner of vaginal moisturizers or polycarbophil gels [53]. Silicone-based products (e.g., KY Liquibeads®) may last longer than either water- or glycerin-based products. The ideal combination is to insert polycarbophil gels intravaginally 4–7 times per week, and utilize generous amounts of a glycerin-based vaginal lubricant before and during sexual activity [5,7,8,11,20]. This combination may not reverse vaginal atrophy, but may provide additional short-term comfort during sexual activity.

## 7. Estrogen Therapy

Vaginal hormone therapies are viable options for women experiencing atrophic vaginitis without a known history of breast cancer, although their safety in breast cancer patients continues to be debated [54,55,56,57,58,59,60,61,62,63]. The level of estrogen absorption is variable [7,22,23,24,61,62,63,64,65] which raises concern for women with breast cancer that were previously treated for estrogen dependent tumors. Much of the treatment of breast cancer relies on the reduction and virtual elimination of estrogen levels through chemotherapy-induced ovarian suppression and oral hormonal agents such as aromatase inhibitors. Therefore, even a small rise in serum estrogen is of concern that circulating estrogen may lead to or promote higher risks of cancer recurrence [36,54]. Consequently, systemic therapies are usually avoided in breast cancer patients because the potential risks are thought to outweigh the benefits [54].

The use of vaginal hormone products, safety profiles, and levels of systemic bioavailability and absorption in breast cancer patients remains understudied. Large, prospective randomized control studies measuring the safety of vaginal estrogen therapy have not been performed. Nevertheless, when the decision is made to use vaginal hormonal products in a breast cancer survivor, it is important to fully dose the woman at the onset in order to heal vaginal tissues (e.g., at least three months) and provide maintenance therapy with the lowest dose possible after tapering from the initial full dose [7].

### 7.1. Systemic Estrogen Therapy

When non-hormonal lubricants and moisturizers are insufficient for managing painful symptoms, exogenous hormonal therapies aim to replace deficient estrogen with systemic or local pharmaceutical interventions [54,55,56,57]. Systemic estrogen-based therapies are the most studied means of alleviating menopausal symptoms [7,24,54], delivered in oral pills or transdermal patches. Women can expect up to a 75% reduction in frequency and 87% reduction in severity of symptoms when prescribed systemic estrogen [55,56].

### 7.2. Oral Hormones in Women with Breast Cancer

The HABITS trial studied the effects of systemic hormone replacement therapy on breast cancer recurrence among Scandinavian breast cancer survivors (N = 447) [59]. Hormonal therapies were administered as combined or sequential estradiol hemihydrates and norethisterone acetate. The study closed prematurely due to reports of an increased risk of breast cancer recurrence [59] among women taking systemic estrogen. A four-year follow-up of the study sample found that women in the hormone replacement therapy group had twice the rate of a breast cancer event [59] as compared to the control group (HR = 2.4).

The Stockholm trial studied breast cancer survivors (N = 378) randomized to hormonal therapy or non-hormonal therapy [66]. Hormonal therapy included cyclic estradiol and medroxyprogesterone acetate or estradiol valerate alone [66]. The trial, similar to HABITS, prematurely ceased due to safety concerns of breast cancer recurrence [66,67]. In contrast to the HABITS trial, the Stockholm trial did not actually find an increase in breast cancer recurrence after a median follow up period of 4.1 years in the hormone replacement study arm [66]. Tibolone is a synthetic steroid that converts into three metabolites that bind to estrogen receptors in the vagina [68]. In a non-randomized, open-label study of postmenopausal women (N = 113), the use of tibolone over six years reversed vaginal atrophy and improved symptoms [68].

A prospective randomized placebo controlled study was conducted to evaluate the safety of tibolone in breast cancer survivors (N = 3100) [69]. The trial was terminated early due to an increase in breast-cancer related events in the tibolone arm (15.2%) as compared to the placebo arm (10.7%) [69].

### 7.3. Local Estrogen Therapy

Strong evidence exists and is endorsed by The North American Menopause Society and International Menopause Society [24] that local estrogens in healthy women may be more effective for symptoms of atrophic vaginitis than oral, systemic treatment and may require lower dosing, especially in the treatment of atrophic vaginitis [24,57,58]. Vaginal hormonal products are the most effective and sole intervention for menopausal symptoms limited to vaginal atrophy [7,18,20,22,24] versus oral hormone replacement therapy [59]. Local therapies consist of estradiol preparations and conjugated estrogens inserted intra-vaginally. These preparations are available in many forms including estrogen based vaginal creams, 17β estradiol-releasing intravaginal tablets, estriol containing pessaries, estradiol releasing vaginal rings, and estrogen-embedded intrauterine devices [54,58]. All forms of local estrogen therapies have shown similar rates of effectiveness, although they have varying levels of systemic absorption [7,14,18,22,23,24,25,46,54,55,56,57,58]. Systemic absorption rates may differ among products, which is an important concept to understand in regard to their use in breast cancer survivors.

Estrogen, estradiol, estriol, and estrone topical formulations can vary in the degree of bioavailability and systemic absorption. Conjugated estrogen is metabolized through the liver and results in increased systemic bioavailability with high serum concentrations [7]. Conjugated estrogen cream has the highest rate of systemic absorption [3,7] of all local vaginal therapies. Therefore, its use should be avoided in women with breast cancer [3,7,59]. Estriol has the lowest potency and cannot be transformed into estradiol or estrone [3,7], nor does estriol cause endometrial proliferation [3,7], another measure of systemic absorption.

### 7.4. Vaginal Hormones in Breast Cancer

Kendall *et al.* [61] measured serum estrogen levels in postmenopausal women (N = 6) with a history of breast cancer who were taking AIs and using 25 mcg estradiol vaginal tablets for severe symptoms of atrophic vaginitis. Serum estradiol levels were measured at baseline, two weeks, four weeks, between seven and ten weeks, and greater than 12 weeks after initiation of therapy [61]. A significant rise in serum estradiol (e.g., from ≤ 5 pmol/L to 72 pmol/L) was found at two weeks, although at four weeks most serum levels dropped to less than 35 pmol/L [61]. Therefore, potential high and persistent serum estradiol levels were of concern. It was unknown if the fragile and thinned vaginal lining initially enabled systemic uptake and then decreased with mucosal healing. It was also unknown if the increase in serum estrogen reversed the estrogen suppression effect from AI treatment [61].

Wills *et al.* [36] conducted a prospective clinical trial of postmenopausal women (N = 24) with a history of estrogen receptor positive breast cancer or with significant risk factors for breast cancer development; both groups were taking AIs or SERMs. Participants used either a 25 mcg estradiol vaginal tablet or ring for three months; the control group had no hormone-containing vaginal therapy [36]. Serum estradiol samples were obtained from all participants at three months. The researchers found that both the intravaginal estradiol ring and tablet users, despite long term usage, had elevated circulating estradiol levels [36], and the researchers argue that these elevated levels occurred even with cornification of tissues [36].

Labrie *et al.* [64] measured serum estradiol levels in postmenopausal women (N = 20) after seven consecutive days of treatment with 25 mcg estradiol vaginal tablets or 0.625 mg conjugated estrogen vaginal cream. A fivefold increase in serum estradiol was present after one week indicating systemic uptake of the intravaginal estrogens [64].

A retrospective study was conducted of women with breast cancer (N = 1472); 4.7% (n = 69) of these women were using low-dose 25 ug estradiol-containing vaginal tablets or 0.5 mg estriol cream for symptoms of atrophic vaginitis [65]. An increased risk of breast cancer recurrence was not found in this group after an average follow-up of 5.5 years as compared to number of recurrences in the control group [65].

In a prospective, randomized study of 10 postmenopausal women with breast cancer and taking AIs, a two-week span of daily 0.5 mg vaginal estriol did not increase serum estrogen or estradiol levels [3]. The use of estriol is of promise in breast cancer survivors given the minimal bioavailability and systemic uptake of the drug [3].

The use of local hormonal therapy is theoretically contraindicated, although a retrospective, nested case-control study of women with breast cancer (N = 13,479) that used concomitant tamoxifen (n = 10,806) or AIs (n = 2673) and local estrogen was conducted [67]. Overall, the risk of recurrence with local hormonal therapy was not increased as compared to the control group (RR: 0.78, 95% CI, 0.48–1.25) [67]. In stratified analyses, the risk was likewise not increased in those women on tamoxifen (RR: 0.83, 95% CI, 0.51–1.34) [67]. In women taking AIs, the risk was not estimable as no women experienced a recurrence [67].

The North American Menopause Society 2013 Position Statement supports that topical vaginal estrogen can be prescribed to breast cancer survivors with estrogen/progesterone negative tumors [24]. To date, there is no data that specifically separates groups of ER+PR+ or ER-PR- tumors in studies of the effectiveness, feasibility, or safety of estrogen in these groups.

### 7.5. Promestriene

Promestriene is an analogue of estradiol with minimal systemic absorption. Promestriene as a topical agent is effective in reducing the side effects of urogenital atrophy [70] that continues under study.

## 8. Emerging Therapies for Women with Breast Cancer

### 8.1. Bazedoxifene

Emerging studies provide data for consideration of bazedoxifene combined with conjugated equine estrogens (BZA/CE) for FDA-approval to treat symptoms of postmenopausal vulvovaginal atrophy [71]. As a single agent, bazedoxifene alone is not effective in relieving symptoms; together, these drugs are very efficacious in symptom relief of vulvovaginal atrophy in postmenopausal women [71]. It remains unknown if this combination will be safe and well tolerated in women with breast cancer [71].

### 8.2. Androgens

Few data are available in the use of vaginal testosterone in breast cancer although the vulva and vagina have androgen receptors. Testosterone can induce proliferation of the vaginal epithelium but testosterone’s conversion to estrogen is blocked by aromatase inhibition and therefore may be effective in reversing atrophic changes without raising circulating estrogen levels and compromising aromatase inhibitor therapy [72].

Witherby *et al.* [72] assessed the use of daily vaginal testosterone to treat vaginal atrophy in women with breast cancer receiving AIs. Testosterone cream in one of two dosages, 150 mcg (n = 10) or 300 mcg (n = 10) was applied to the inner labia minora, introitus, and internal vaginal mucosa for 28 days [72]. Both dosages of testosterone improved symptoms of vaginal atrophy including dyspareunia (*p* = 0.001) and vaginal dryness (*p* < 0.001), although only the 300 mcg decreased vaginal pH (e.g., 5.5–5.0), and improved the vaginal maturation index (e.g., 20%–40%) [72]. No significant changes in serum estradiol levels (*p* = 0.91) were observed [72]. Testosterone levels increased from < 21 ng/dL at baseline to ≤ 42 ng/dL at 28 days; one participant had a testosterone level of 113 ng/dL [72].

### 8.3. Dehydroepiandrosterone

(DHEA) has also been studied as an intervention for atrophic vaginitis. Preclinical data demonstrated that DHEA converts to androgens in the vaginal lamina propria [73,74] and binds to both estrogen and androgen receptors. A phase III study of postmenopausal women suffering from vaginal atrophy (N = 218) measured the effect of three different doses of daily intravaginal DHEA for 12 weeks: 0.25, 0.50, or 1.0% [73]. Labrie *et al.* suggested DHEA exerts benefits on all three layers of the vagina, e.g., the superficial epithelium, the lamina propria and the muscularis [73,74]. These effects strengthened the vaginal wall and reversed vaginal atrophy without increase of circulating sex steroid levels [73,74]. The 0.5% DHEA was found to be the most effective with significant decreases in vaginal pH and parabasal cells, increase in superficial cells, and improvement in vaginal atrophy symptoms [73,74]. Results are consistent with a previous phase II study of one week of DHEA treatment that resulted in an increased maturation value of epithelial cells and decreased pH without increases in serum estrogen [73,74]. Concern remains that DHEA and testosterone are precursors for estrogenic compounds via aromatization [36].

### 8.4. Olive Oil, Vaginal Exercise, and Moisturizer

The OVERcome study (Olive Oil, Vaginal Exercise, and Moisturizer) measured the effects of novel, non-hormonal interventions on dyspareunia and other sexual problems in women with breast cancer [75]. OVERcome resulted in significant improvements in quality of life, sexual function, and dyspareunia (*p* < 0.001) [75]. Maximal benefits were noted after 12 weeks of intervention [75].

### 8.5. Vaginal pH-Balance Gel

A study of breast cancer survivors (N = 88) measured the effect of a balanced pH vaginal gel as compared to placebo [76]. The pH-balanced gel provided significant (*p* = 0.001) improvements in vaginal dryness and dyspareunia as compared to placebo, and was effective in reducing the vaginal pH (*p* < 0.001*)* [76]*.* In addition, the pH-balanced gel enhanced vaginal maturation index (*p* < 0.001) and vaginal health index (*p* = 0.002) [76]. No significant difference in adverse events between the two gels was noted [76].

## 9. Therapies with Unknown Benefit Undergoing Further Investigation

### Ospemifene

Ospemifene is a novel tissue-selective estrogen agonist/antagonist for the treatment of vulvar and vaginal atrophy with symptoms of dyspareunia as approved by the U.S. Food and Drug Administration [77,78,79,80,81,82]. Ospemifene, is the first non-estrogenic treatment for dyspareunia, and provides an alternative to oral or local estrogen therapies [77]. This preparation is provided in a once-daily oral 60 mg tablet [77]. Ospemifene is similar to tamoxifen with several pre-clinical and clinical studies to support its use [77]. Ospemifene provides an estrogenic effect on the vulvo-vaginal cells by increasing the proportion of superficial cells in vaginal mucosa, reducing the number of parabasal cells, and decreasing vaginal pH [77].

Several studies have substantiated the positive effect of ospemifene on atrophic vaginitis [78]. In a placebo-controlled, randomized Phase II trial (N = 160), three doses of ospemifene were tested: 30, 60, and 90 mg per day, or placebo [78]. Women in all three ospemifene groups experienced a positive effect on vaginal epithelial cells [78]. Another Phase II trial (N = 118) compared ospemifene to raloxifene and their effect on the genital tract [79]. Ospemifene had a positive estrogenic effect with epithelial changes in the vagina, as compared to raloxifene which showed no changes from baseline [79]. A placebo-controlled study (N = 826) across 76 centers studied 30 or 60 mg per day ospemifene or placebo [80]. Results were significant for increases in vaginal epithelial cells, decreases in parabasal cells, improvements in vaginal maturation index, and decrease in pH in both ospemifene groups [80]. The second placebo-controlled study (N = 605) across 110 sites compared 60 mg of ospemifene to placebo, with findings similar to the other Phase III trial [81]. Although preclinical models of breast cancer with ospemifene have been favorable, there is currently no safety data for ospefimene in patients with history of breast cancer.

## 10. Summary

The decision to use vaginal hormonal therapy must be made on an individual basis with discussions between the treating physician and the patient. Discussion and understanding of the pros and cons of vaginal hormone therapy are critical prior to their use in women with and without breast cancer. Contradictory outcomes among several studies that investigated topical hormonal therapy to treat atrophic vaginitis have resulted in controversy over the safe use of topical therapies and remain open to debate. Some studies suggested that serum estrogen levels were increased by topical estrogen therapies and were contraindicated in breast cancer survivors, while other studies proposed there was no significant threat to the safety of patients secondary to limited or no increase in serum levels. No definite recommendations exist that physicians and patients can rely on in regard to local hormonal treatment to alleviate severe symptoms of atrophic vaginitis. More data is needed to confirm the systemic effects of topical vaginal estrogen.

Multiple studies in postmenopausal women with ospefimine have demonstrated superior benefits to the vaginal epithelium, maturation index, pH, and decreases in parabasal cells earning FDA approval for dyspareunia in atrophic vaginitis. Ospefimine is a non-hormonal estrogen agonist/antagonist similar to tamoxifen. These studies provide promising results for women with breast cancer given the pharmaceutical configuration of the selective estrogen response modifier. However, additional investigation is warranted to ensure safety in the breast cancer population.

## 11. Conclusions

Atrophic vaginitis is a condition that impairs the quality of life of breast cancer patients. Continued investigations of various treatments for atrophic vaginitis are necessary; estrogen, DHEA, testosterone, and pH-balanced gels continue to be evaluated in ongoing studies. Definitive results are needed pertaining to the safety of topical estrogens in breast cancer survivors. Due to several contradictions in published studies, a large, randomized, placebo controlled study investigating changes in serum levels of estrogen from varying doses and forms of topical vaginal estrogen therapies is warranted. In addition, further studies are needed to develop safe and effective non-hormonal treatment options for atrophic vaginitis. Finally, continued trials of testosterone, DHEA, and pH-controlled gels are desired to fully understand the safety and efficacy behind each therapy as a treatment for atrophic vaginitis in breast cancer patients. Perhaps the application of non-hormonal therapies *prior* to the development of severe atrophic vaginitis may decrease the degree of discomfort and permanent changes. Duration of therapy with all of these therapies is currently unknown and at this time, we recommend individualizing duration of therapy based on symptom improvement and quality of life. If there is a significant improvement in symptom burden, the therapy can be administered as needed and if symptoms continue, more continued therapy would be advisable. With the advent of successful therapy for atrophic vaginitis in the breast cancer patient population, the lives of many can be improved through reduced side effects.
